# From Defense to Disease: How the Immune System Fuels Epithelial–Mesenchymal Transition in Ovarian Cancer

**DOI:** 10.3390/ijms26094041

**Published:** 2025-04-24

**Authors:** Michał Kos, Paulina Mertowska, Sebastian Mertowski, Jacek Roliński, Aleksandra Krasińska-Płachta, Tomasz Urbanowicz, Marek Gogacz, Ewelina Grywalska

**Affiliations:** 1 II Clinic of Gynecology, Medical University of Lublin, 20-093 Lublin, Poland; michal.kos@mp.pl (M.K.); marek.gogacz@umlub.pl (M.G.); 2 Department of Experimental Immunology, Medical University of Lublin, 20-093 Lublin, Poland; sebastian.mertowski@umlub.pl (S.M.); ewelina.grywalska@umlub.pl (E.G.); 3 Department of Clinical Immunology, Medical University of Lublin, 4a Chodźki Street, 20-093 Lublin, Poland; jacek.rolinski@umlub.pl; 4 Department of Ophthalmology, Poznan University of Medical Sciences, Fredry 10, 61-107 Poznan, Poland; aleksandra.krasinska@usk.poznan.pl; 5 Cardiac Surgery and Transplantology Department, Poznan University of Medical Sciences, 61-848 Poznan, Poland

**Keywords:** EMT, cancer, oncology, immune system, prognostic factors, oncogenesis

## Abstract

Ovarian cancer is one of the most deadly gynecological cancers, with over 300 thousand new cases per year, most of which are diagnosed in advanced stages. The limited availability of effective biomarkers and lack of characteristic symptoms make early diagnosis difficult, resulting in a five-year survival rate of 30–40%. Mutations in the *BRCA1* and *BRCA2* genes and abnormalities of signaling pathways such as PI3K/AKT and TP53 play a key role in the progression of ovarian cancer. The immune system, which can act against tumors, often supports tumor development in the ovarian cancer microenvironment through immunoevasion, which is influenced by cytokines such as IL-6, IL-10, and TGF-β. Epithelial-to-mesenchymal transition (EMT) allows cancer cells to acquire mesenchymal characteristics, increasing their invasiveness and metastatic capacity. Immunological factors, including pro-inflammatory cytokines and signals from the tumor microenvironment regulate the EMT process. This review aims to present the role of EMT in ovarian cancer progression, its interactions with the immune system, and potential biomarkers and therapeutic targets. Modulation of the immune response and inhibition of EMT may constitute the basis for personalized therapies, which opens new possibilities for improving the prognosis and efficacy of treatment in patients with ovarian cancer.

## 1. Introduction

**Figure 1:**
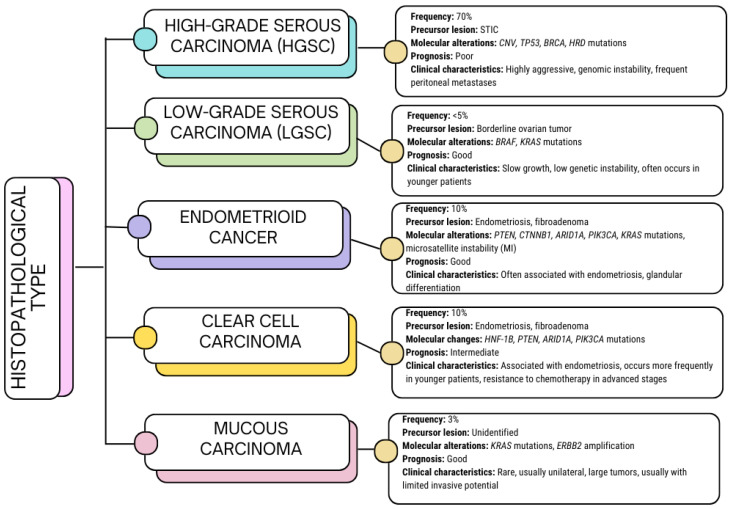
Graphical representation of histopathological types of ovarian cancer based on [8,9,10,11].

Ovarian cancer is a significant health challenge worldwide, being the eighth most commonly diagnosed malignancy in women and the leading cause of gynecological cancer deaths in developed countries [1,2,3]. Global epidemiological data indicate that more than 300,000 new cases of ovarian cancer are diagnosed each year, of which approximately 70% are detected in the advanced stage (III/IV according to the International Federation of Gynecology and Obstetrics—FIGO classification) [4,5]. This late diagnosis is mainly due to the lack of specific symptoms in the early stages of the disease and the limited availability of sensitive and specific diagnostic biomarkers. The five-year survival rate of patients with ovarian cancer is only 30–40%, making it one of the most deadly gynecological cancers [6,7]. In terms of histopathological classification, ovarian cancer is divided into several subtypes, of which the most common, high-grade serous carcinoma (HGSC), accounts for approximately 70% of cases. Other subtypes, such as endometrioid, mucinous, or clear-cell carcinoma, differ in molecular, etiological, and clinical terms. Modern molecular classification additionally emphasizes the role of mutations in the *BRCA1* (breast cancer gene 1) and *BRCA2* (breast cancer gene 2) genes and disturbances in signaling pathways such as PI3K/AKT (phosphoinositide 3-kinases/protein kinase B), RAS/MAPK (rat sarcoma/mitogen-activated protein kinase) or TP53 (tumor protein P53), which are key to the progression of this cancer (Figure 1) [8,9,10,11].

The importance of the immune system in the pathogenesis of ovarian cancer is becoming increasingly evident. The immune system plays an ambiguous role: it can either eliminate cancer cells or support their survival and progression. In the ovarian cancer microenvironment, cytotoxic T lymphocytes (CTLs) are associated with a favorable prognosis. In contrast, the accumulation of immunosuppressive cells, such as regulatory T lymphocytes (Tregs), tumor-associated macrophages (TAMs), or myeloid-derived suppressor cells (MDSCs), promotes tumor immunoevasion. Additionally, cytokines such as IL-6, IL-10, and TGF-β (transforming growth factor-β) modulate the activity of the immune system in a way that promotes tumor growth [12,13,14,15,16].

One of the key molecular mechanisms underlying ovarian cancer progression is epithelial–mesenchymal transition (EMT). This process allows cancer cells to lose epithelial characteristics, such as cell polarization and tight junctions, and to acquire mesenchymal properties, including the ability to migrate and invade. EMT is strongly regulated by immunological factors, including pro-inflammatory cytokines (e.g., TNF-α, IL-6) and signals from the tumor microenvironment, such as TGF-β and chemokines. Communication between cancer cells and the immune system can initiate EMT and support its maintenance, leading to increased tumor invasive and metastatic capacity [17,18].

Therefore, this literature review aimed to present information regarding the understanding of the role of the immune system in regulating EMT and its interaction with the tumor microenvironment, which is crucial for identifying new prognostic biomarkers and therapeutic targets. This knowledge opens new possibilities in the personalization of ovarian cancer therapy, including strategies based on the modulation of the immune response and inhibition of EMT.

## 2. Ovarian Cancer Microenvironment: Complexity of Structure and Function

The immune system plays a multifaceted and complex role in the pathogenesis of ovarian cancer, combining protective functions with mechanisms supporting the development of the tumor. On the one hand, it acts as a key agent in immunosurveillance, identifying and eliminating cancer cells. This phenomenon is based on the recognition of tumor antigens by T lymphocytes and the activation of the effector response, in which cytotoxic mechanisms of the immune system destroy cancer cells. On the other hand, as the disease progresses, cancer cells gain the ability to modulate the immune response, creating an environment conducive to tumor survival and progression. This phenomenon includes changes in the tumor microenvironment and the activation of specific mechanisms that evade the immune response, known as immunoevasion. The balance between immune surveillance and immunoevasion affects the dynamics of ovarian cancer development and the effectiveness of therapy, including immunotherapy [19,20,21,22].

Ovarian cancer is characterized by high immunogenicity, which means the ability to induce an immune response. Cancer cells expose cancer antigens on their surface, such as CA125 (MUC16) (cancer antigen 125), HE4 (human epididymis protein 4), WT1 (Wilms’ tumor 1), NY-ESO-1 (New York esophageal squamous cell carcinoma 1), or mesothelin, which T lymphocytes can recognize. These antigens are presented to T lymphocytes by MHC class I molecules (major histocompatibility complex), which initiates the activation of an anticancer response. Despite this, ovarian cancer cells have developed mechanisms that allow them to avoid immune elimination. One of the most important mechanisms is subversion and coercion, which consists in suppressing the immune response using the expression of inhibitory ligands, such as PD-L1 (programmed cell death ligand 1), which binds to the PD-1 (programmed cell death protein 1) receptor on T lymphocytes, leading to their silencing. Similarly, interactions with CTLA-4 (cytotoxic T lymphocyte-associated antigen 4) receptors on T lymphocytes result in a decrease in their effector activity. Additionally, cancer cells secrete immunosuppressive cytokines, such as IL-10 and TGF-β, which inhibit the effector functions of T lymphocytes and support the development of a population of regulatory lymphocytes (Tregs), which play a key role in suppressing the antitumor response [23,24,25,26,27,28,29].

Another process that plays a key role in ovarian cancer progression is immunoediting. This process consists of three stages: elimination, equilibrium, and escape. In the elimination phase, the immune system effectively recognizes and destroys cancer cells. The equilibrium phase is characterized by maintaining a dynamic balance between the immune system and the survival of a part of the cancer cells that can adapt to immune pressure. In the final escape phase, cancer cells acquire the ability to effectively evade immune surveillance by reducing the expression of MHC class I molecules, which limits their recognition by T lymphocytes, or by actively inducing an immunosuppressive tumor microenvironment [30,31,32].

Immunoediting in ovarian cancer is facilitated by interactions between the tumor and its microenvironment. The tumor microenvironment (TME) contains numerous tumor-promoting elements, including TAMs, Tregs, MDSCs, and low oxygen concentration (hypoxia), enhancing angiogenesis and promoting immune tolerance. These complex mechanisms create a highly immunosuppressive environment that favors tumor cell survival, migration, and metastasis, ultimately hampering an effective antitumor response. In ovarian cancer, the TME is a highly complex and dynamic structure consisting of various cell types, a network of blood and lymphatic vessels, the extracellular matrix (ECM), and several biochemical mediators. Consequently, it plays a fundamental role in the pathogenesis of ovarian cancer, determining tumor growth, progression, metastasis, and response to treatment. The microenvironment’s structure, function, and molecular interactions are crucial to understanding the mechanisms underlying immunosuppression, which enables tumors to evade immune elimination while promoting their aggressiveness [33,34,35].

**Figure 2:**
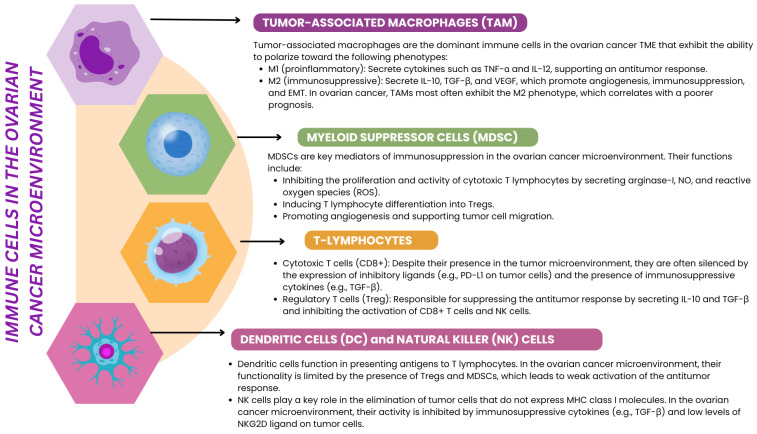
Schematic presentation of the role of immune cells in the ovarian cancer microenvironment, based on [33,34,35].

The ovarian cancer microenvironment comprises three significant components: stromal cells, immune cells, and extracellular matrix components. Stromal cells include cancer-associated fibroblasts (CAFs), pericytes, and endothelial cells, which cooperate to remodel the ECM, stimulate angiogenesis, and support tumor cell migration. Immune cells, such as tumor-associated macrophages (TAMs), myeloid suppressor cells (MDSCs), T cells, B cells, NK cells, mast cells, and dendritic cells, play a key role in modulating the immune response, often acting to the benefit of the tumor by suppressing the antitumor response (Figure 2). The extracellular matrix, composed of collagen, fibronectin, proteoglycans, and remodeling enzymes such as matrix metalloproteinases (MMPs), provides a physical and biochemical structure that supports cancer cell survival and invasion.

Tumor-associated fibroblasts (CAFs) are key stromal elements in the ovarian cancer microenvironment. Their primary role is to remodel the ECM, which promotes tumor cell migration, and to support angiogenesis by secreting growth factors such as TGF-β, VEGF, and HGF. CAFs also promote the epithelial–mesenchymal transition (EMT) process, which allows tumor cells to acquire a more invasive and therapy-resistant phenotype. In the context of immunosuppression, CAFs secrete cytokines such as TGF-β and IL-6, which suppress cytotoxic T cell activity while supporting the development of regulatory T cell (Treg) populations, which are crucial for suppressing antitumor response. In addition, CAFs recruit M2 phenotype macrophages and myeloid suppressor cells (MDSCs), thereby enhancing the immunosuppressive environment that favors tumor survival and progression [36,37,38,39].

The extracellular matrix (ECM) plays a key role in the ovarian cancer tumor microenvironment, being a dynamic and multifunctional component that supports both mechanical and biochemical interactions between tumor cells and the stroma. ECM components, such as collagen, fibronectin, elastin, laminin, and proteoglycans, provide structural support for tumor cell proliferation and migration while regulating intercellular signaling. The ECM provides a physical platform that allows tumor cells to adapt to changing microenvironmental conditions, including hypoxic and immunological stress [39,40,41].

**Figure 3:**
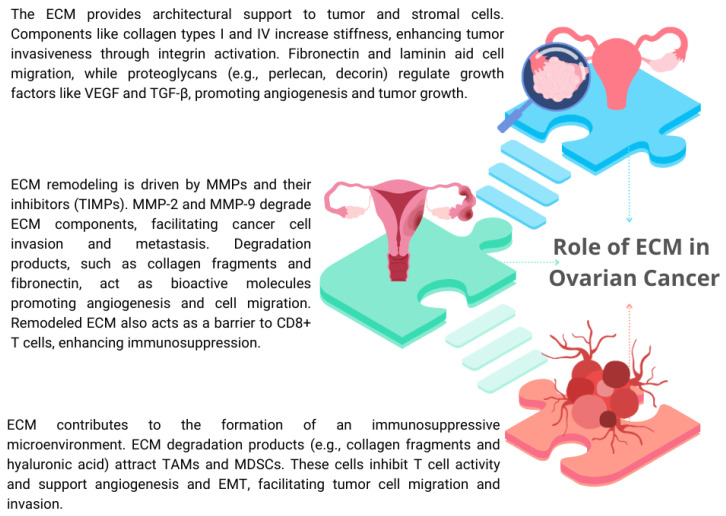
Role of ECM in ovarian cancer, based on [40,41,42].

In healthy tissues, the ECM is a precisely organized network composed of structural proteins such as collagen and elastin, proteoglycans such as perlecan and aggrecan, glycosaminoglycans such as hyaluronic acid and heparin, and adhesive glycoproteins such as fibronectin and laminin. In ovarian cancer, there are significant changes in the composition and organization of the ECM that support the aggressive nature of the disease. One of the key processes is the excessive accumulation of collagen, especially type I, III, and IV, and fibronectin, which leads to increased tumor stiffness. This phenomenon promotes the migration and invasion of cancer cells. At the same time, reduced matrix elasticity is the result of dysregulation of the balance between ECM-degrading enzymes such as metalloproteinases (MMPs) and their natural inhibitors (TIMPs) [40,41,42] (Figure 3).

The ECM plays a key role in regulating tumor growth through interactions with integrin receptors on the surface of tumor cells. For example, integrins α5β1 and αvβ3 stimulate tumor cell proliferation by activating the PI3K/AKT and MAPK signaling pathways. These pathways promote cell survival and division, making the ECM a key element of the tumor microenvironment. Changes in the ECM also promote tumor cell migration and invasion. Enzymatic activity of MMP-2 and MMP-9 enables degradation of the basement membrane, mainly collagen IV, which facilitates the penetration of tumor cells into the surrounding tissues. Additionally, the ECM creates so-called “collagen pathways” that promote tumor cell migration. In metastasis, the key role is played by the increased expression of hyaluronic acid and its receptors, such as CD44, which promote the adhesion of cancer cells to the peritoneum and other organ surfaces. The ECM also participates in of premetastatic niches, i.e., microenvironments conducive to the settlement of metastases. Interactions between the ECM and cancer cells also affect the efficacy of therapy. Dense and stiff ECM constitutes a mechanical barrier to the penetration of anticancer drugs. In addition, pro-survival signaling induced by the ECM, mainly through integrins, can lead to resistance to chemotherapy by activating pathways such as PI3K/AKT. Moreover, excessive ECM production limits angiogenesis within the tumor, creating a hypoxic environment that selects cells more resistant to treatment [40,41,42,43,44,45].

**Figure 4:**
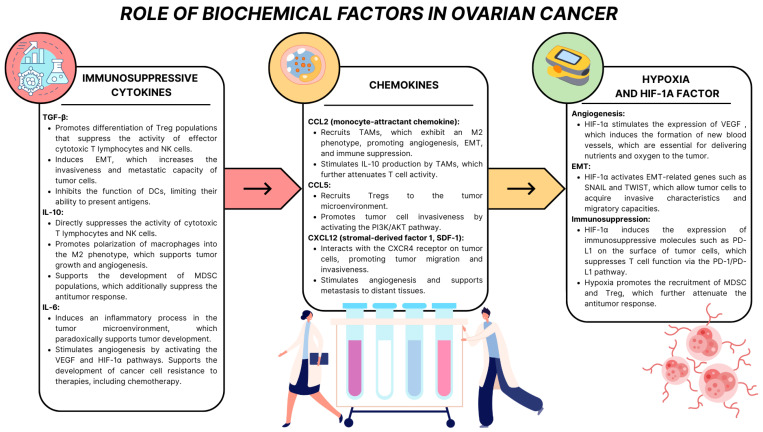
Role of biochemical factors in ovarian cancer, based on [49,50,51,52,53,54,55,56,57,58,59,60,61].

The ovarian cancer tumor microenvironment is characterized by the presence of many biochemical factors that play a key role in the development, progression, and ability of the tumor to evade the immune response. These factors, including cytokines, chemokines, remodeling enzymes, growth factors, and hypoxia-inducible molecules, form a complex signaling network that regulates the interactions between tumor, stromal, and immune cells (Figure 4). Their actions are directed at supporting the proliferation, migration, and adaptation of tumor cells to the changing conditions of the microenvironment while suppressing an effective antitumor immune response [46,47,48].

Immunosuppressive cytokines such as TGF-β, IL-10, and IL-6, secreted by cancer cells, CAFs, and TAMs, modulate the immune system, leading to its suppression and promotion of tumor development. TGF-β acts in multiple ways, promoting the differentiation of T lymphocytes into regulatory populations (Tregs), which suppress the activity of effector cytotoxic T lymphocytes and NK cells, and promoting EMT, which increases the invasiveness and metastasis of cancer cells. TGF-β also inhibits the function of dendritic cells (DCs), limiting their ability to present antigens and attenuating the adaptive response. IL-10, secreted mainly by TAMs, supports immunosuppression by inhibiting the proliferation and activity of cytotoxic T lymphocytes while promoting the development of the M2 macrophage phenotype, which is associated with angiogenesis, EMT, and tumor growth. IL-6, activating the JAK/STAT3 pathway, stimulates inflammatory processes in the tumor microenvironment, which paradoxically support tumor development, promote angiogenesis, and support the adaptation of tumor cells to therapy, including chemotherapy [49,50,51,52,53].

One of the most studied chemokine pathways in ovarian cancer is the CXCL12/CXCR4 axis. CXCL12, also known as SDF-1 (stromal-derived factor 1), is a potent chemoattractant whose receptor CXCR4 is overexpressed on the surface of ovarian cancer cells. Interaction between CXCL12 and CXCR4 leads to the activation of signaling pathways such as PI3K/AKT and MAPK, which promote cancer cell proliferation, survival, and migration. In addition, CXCL12 attracts cancer cells to tissues rich in this ligand, which facilitates metastasis. Studies have shown that CXCL12 also promotes angiogenesis by stimulating the formation of new blood vessels that supply the tumor with oxygen and nutrients. Another important pathway is the CXCL8/CXCR1/CXCR2 axis. CXCL8 (interleukin 8) is a chemokine with potent proangiogenic and pro-inflammatory properties. CXCL8 promotes blood vessel formation in ovarian cancer by activating CXCR1 and CXCR2 receptors on endothelial cells. This chemokine also acts as a factor stimulating cancer cell migration and invasion. Its presence in the tumor microenvironment correlates with an aggressive tumor phenotype, increased invasiveness, and poorer prognosis for patients. Furthermore, CXCL8 can regulate the activity of MMPs, such as MMP-2 and MMP-9, which degrade the extracellular matrix, allowing cancer cells to invade neighboring tissues. Chemokines also play an essential role in modulating the tumor microenvironment by influencing the immune system. CXCL12 attracts MDSCs and regulatory T cells (Tregs), which inhibit the activity of effector T cells, attenuating the antitumor response. Creating an immunosuppressive microenvironment promotes tumor growth and its resistance to immunotherapy. Another example is the effect of CXCL8 on the recruitment of neutrophils and M2 macrophages, which support tumor progression by secreting growth factors and proteases [54,55,56,57].

Hypoxia, which results from limited blood supply in a rapidly proliferating tumor, plays a key role in creating an immunosuppressive microenvironment. Oxygen deficiency in the tumor microenvironment leads to the activation of adaptive mechanisms that increase tumor aggressiveness, support its invasiveness and metastasis, and affect resistance to treatment. Oxygen deficiency activates HIF-1α (hypoxia-inducible factor 1-alpha), which induces VEGF expression, supporting angiogenesis, and activates EMT-related genes such as *SNAIL* and *TWIST*, which increase tumor cell invasiveness. HIF-1α also stimulates the expression of immunosuppressive molecules such as PD-L1, which bind to the PD-1 receptor on T lymphocytes, suppressing their function and effectively inhibiting the antitumor response. Moreover, hypoxia promotes the recruitment of MDSCs and Treg lymphocytes, which enhance the suppression of effector functions of immune cells. The central element of the cancer cell response to hypoxia is HIF-1α, which increases the expression of genes responsible for angiogenesis, cell survival, and metabolic adaptation. One of the most important effects of HIF-1α activation is the increased production of proangiogenic factors such as VEGF, which stimulates the formation of new blood vessels supplying the tumor with oxygen and nutrients but at the same time contributes to the irregularity and dysfunction of the vessels in the tumor. Hypoxia significantly promotes the resistance of ovarian cancer cells to treatment, as it changes the metabolism of cancer cells, increasing their dependence on glycolysis and leading to acidification of the tumor microenvironment. This metabolic change reduces the efficacy of chemotherapy and limits the availability of drugs in the tumor. Hypoxia also induces the expression of antiapoptotic proteins, which protect cells from therapy-induced death. In the context of immunotherapy, hypoxia also weakens the activity of immune cells such as T lymphocytes and NK cells, weakening the antitumor response. Moreover, it stimulates macrophage recruitment and polarization toward the M2 phenotype, which supports tumor growth, suppresses the immune response, and promotes angiogenesis. Hypoxia also induces EMT, which increases the invasiveness of tumor cells and their ability to metastasize. Under hypoxic conditions, the expression of MMPs is also increased, which degrades the extracellular matrix, facilitating tumor cell invasion into neighboring tissues [58,59,60,61].

## 3. EMT in Ovarian Cancer

EMT is a dynamic biological process in which epithelial cells lose their characteristic features, such as intercellular adhesion and polarity, to acquire mesenchymal features, such as the ability to migrate, invasiveness, elasticity, and increased resistance to apoptosis. EMT plays a key role in the development and progression of ovarian cancer, supporting its aggressiveness, metastatic capacity, and resistance to therapies. In ovarian cancer, EMT might allow cancer cells to detach from the primary tumor, migrate in the peritoneal cavity, and implant on the peritoneal membrane and internal organs such as the liver or intestines [17,18,62,63].

Significant changes in the phenotype of cancer cells characterize EMT. During EMT, there is a loss of epithelial features, including a decrease in the expression of markers such as E-cadherin, a key glycoprotein responsible for intercellular adhesion and maintenance of epithelial structure. This is accompanied by a decrease in the level of other epithelial markers, such as occludin and claudin, responsible for tight junctions’ integrity. At the same time, cancer cells gain mesenchymal features, such as the increased expression of markers such as N-cadherin, vimentin, and fibronectin, which support motility and invasiveness. This process also includes the reorganization of the actin cytoskeleton, which allows cell migration and adaptation to the new environment [17,18,62,63].

The function of EMT in oncology is multifaceted. First, EMT enables cancer cells to break away from the primary tumor and migrate in the peritoneal fluid, leading to colonization of the peritoneal membrane and abdominal organs. Cells undergoing EMT gain a greater capacity for invasion due to increased activity of MMPs, which degrade the extracellular matrix, allowing them to penetrate neighboring tissues. EMT also supports metastasis, allowing cells to reach and settle in distant locations. Additionally, EMT leads to more excellent drug resistance, due to the acquisition of antiapoptotic features and changes in metabolism that increase their survival in difficult microenvironmental conditions. In ovarian cancer, EMT is a key mechanism driving disease progression and represents an important target for new therapeutic strategies [17,18,62,63].

### 3.1. Types of EMT

Basing on biological function and the molecular mechanisms involved, EMT can be divided into three main types: EMT type 1, EMT type 2, and EMT type 3, which differ in their biological context, mechanisms, and functions (Table 1).

Oncogenesis in ovarian cancer, especially in the context of HGSC, is closely related to the EMT, which plays a key role in the initiation, progression, and poorer prognosis of patients. One of the characteristic features of the ovarian epithelium is the lack of E-cadherin expression in normal cells, with the simultaneous presence of mesenchymal markers such as vimentin or N-cadherin. This reflects the mesodermal origin of the ovarian surface epithelium, which differs from the respiratory or digestive system epithelium. On the contrary, healthy fallopian tube epithelium and benign and malignant epithelial ovarian tumors show higher expression of E-cadherin [62].

However, in the case of HGSC, a “cadherin switching” phenomenon occurs, consisting of an increased expression of N-cadherin and a simultaneous decrease in E-cadherin. Additionally, the tumor cells coexpress vimentin and cytokeratin, indicating a mixture of epithelial and mesenchymal features. This molecular characteristic is distinct not only from benign tumors but also from other histological subtypes of ovarian cancer. This suggests that EMT plays a direct role in the initiation and progression of HGSC, the most common subtype of EOC cancer [65,66].

Modern research confirms the involvement of EMT in carcinogenesis [67,68]. For example, a transcriptomic study by Xu et al. revealed the presence of EMT-related gene signatures in HGSC cells, such as *NOTCH1*, *SNAI2*, *TGFBR1*, and *WNT11*. These genes were associated with poorer prognosis [69]. In addition, a high percentage of infiltrating immune cells, such as dysfunctional M1 macrophages and CD8+ TRM and TEX cells, have been observed in early-stage tumors. As the disease progresses, CAFs take over and can induce EMT in tumor cell cultures [70]. A study by Deng et al. (2022) confirmed that metastatic lesions are particularly enriched in EMT-related genes, further underlining the role of this process in the spread of ovarian cancer [71].

Among the EMT factors associated with tumorigenesis, ZEB1 is an important repressor of the gene encoding E-cadherin (*CDH1*). Studies by Sohn et al. (2021) have shown that the mesenchymal subtype of HGSC, characterized by EMT gene signatures such as *ZEB1*, *β-catenin*, and *TCF4*, is associated with poorer overall survival compared to the subtype characterized by abnormalities in homologous recombination repair (HRR) mechanisms. Interestingly, no overlap between EMT signatures and mutations in HRR genes was observed, suggesting that currently used HRR-targeted drugs might prove ineffective against the mesenchymal subtype of HGSC [66].

β-Catenin, an essential component of the Wnt pathway, is also engaged in EMT. Mutations in β-catenin are prevalent in the endometrioid subtype of ovarian cancer, the third most common. Activation of the Wnt/β-catenin pathway correlates with poorer prognosis and increased tumor aggressiveness. Furthermore, increased β-catenin activity is associated with decreased E-cadherin levels, which is characteristic of EMT and promotes cancer cell migration and invasion [72,73].

Other histological types of ovarian cancer may also have a significant role for EMT in their pathogenesis, although their relationship to this process is not yet fully understood. One such type is clear-cell ovarian cancer (CCC), a rare subtype of ovarian cancer that is notable for its poor response to platinum-based chemotherapy [74]. More than 50% of CCC cases are associated with mutations in the gene encoding the AR-ID1A protein (AT-rich interactive domain-containing protein 1A). Studies have shown that silencing ARID1A in cell lines leads to the increased expression of EMT markers such as vimentin and N-cadherin. At the same time, NF-κB-dependent PD-L1 suppression was observed, which further emphasizes the role of ARID1A in the modulation of tumor immunosuppression, a mechanism enabling cancer cells to evade immune response [75,76,77].

Ovarian carcinosarcomas, one of the rarest ovarian tumors, represent an interesting case in EMT research due to their unique molecular environment. These rare tumors offer the opportunity to study local interactions between epithelial and mesenchymal phenotype cells, which may shed light on the mechanisms of EMT in oncogenesis [78]. In a recent study by Ho et al., EMT has been shown to play a key role in the pathogenesis of carcinosarcoma [78]. The authors observed that mesenchymal phenotype cells that retained genetic traces of monoclonality with epithelial cells showed a significantly higher expression of EMT-related genes. Moreover, molecular EMT scores were significantly more pronounced in epithelial phenotype carcinosarcoma segments than classical EOC cells. These results support the hypothesis that carcinosarcomas may arise through lineage conversion, in which EMT plays a key role as a mechanism of phenotypic transformation [78].

### 3.2. Molecular Mechanisms of EMT

The molecular mechanisms of EMT in ovarian cancer involve a complex network of intracellular signaling pathways and external stimuli from the tumor microenvironment. The EMT process is driven by dynamic interactions between key transcription factors, such as *SNAIL*, *TWIST*, and *ZEB1*, and major cell signaling pathways, involving, among others, TGF-β, Wnt/β-catenin, Notch, and microenvironmental factors, such as hypoxia, cytokines, and suppressor cells. Together, these mechanisms lead to the reprogramming of tumor cells, which lose characteristic features of the epithelial phenotype, such as intercellular adhesion and polarity, while acquiring mesenchymal features, such as motility, invasiveness, and increased resistance to apoptosis [17,18,79].

Transcription factors play a key role in the regulation of EMT by modulating the expression of genes responsible for cell adhesion and cytoskeletal reorganization. For example, *SNAIL* and *SLUG*, members of the Snail protein family, repress the E-cadherin gene *CDH1*, which leads to the loss of cell–cell adhesion and increased motility of cancer cells. In turn, *ZEB1* and *ZEB2*, by inhibiting E-cadherin and inducing the expression of mesenchymal markers such as vimentin, enhance cell plasticity. *TWIST*, another key regulator of EMT, promotes cytoskeletal reorganization and increases the expression of mesenchymal markers such as N-cadherin, supporting the ability of cancer cells to invade and migrate [80,81,82].

Signaling pathways constitute the molecular basis of EMT, transmitting signals from the tumor microenvironment to transcription factors regulating EMT. TGF-β is one of the main inducers of EMT in ovarian cancer. Activation of TGF-β receptors leads to phosphorylation of SMAD proteins, which initiate the expression of genes promoting EMT in the cell nucleus. Similarly, the Wnt/β-catenin pathway plays an important role in stabilizing β-catenin, which, after translocation to the cell nucleus, activates the expression of genes supporting the mesenchymal phenotype. In turn, the PI3K/AKT and MAPK/ERK pathways support the transition to the mesenchymal phenotype and increase cell survival in difficult microenvironmental conditions [18,72].

Another important pathway is Notch, which regulates cell differentiation and survival. *RUNX1* can modulate the activity of this pathway, influencing the dynamic balance between proliferation and apoptosis of cancer cells. Dysregulation of the Notch pathway is frequently observed in ovarian cancer and is associated with poorer prognosis. The Notch pathway acts synergistically with TGF-β, especially in hypoxic conditions, and supports the expression of mesenchymal markers and proangiogenic factors [83,84].

One of the essential regulators of these processes is the transcription factor *RUNX1*, which participates in the modulation of various signaling pathways. *RUNX1* affects the Wnt/β-catenin pathway, which is crucial for regulating the expression of genes related to cell proliferation and differentiation. Dysregulation of this pathway can lead to uncontrolled growth of cancer cells. In addition, *RUNX1* interacts with the TGF-β pathway, which plays a dual role in carcinogenesis: in the early stages, it acts as a tumor suppressor, inhibiting cell proliferation, while in advanced stages, it can promote invasion and metastasis by inducing EMT [83].

The tumor microenvironment provides extrinsic signals that stimulate EMT and enable its maintenance. Hypoxia, a common event in the ovarian cancer microenvironment, activates HIF-1α, which induces the expression of key EMT genes such as *SNAIL, SLUG*, and *TWIST*, and promotes angiogenesis, increasing the tumor’s capacity for invasion and migration. Cytokines such as IL-6 and CXCL12 activate the JAK/STAT3 and PI3K/AKT pathways, promoting cell phenotypic transition. Suppressor cells such as M2 macrophages and MDSCs secrete EMT-promoting factors such as TGF-β and IL-10, while suppressing the host immune response [49,50,51,52,53].

### 3.3. Immune Cells Involved in EMT in Ovarian Cancer

#### 3.3.1. Macrophages

TAMs play a key role in ovarian cancer progression and metastasis and are among the most abundant immune cells in the tumor microenvironment. TAMs, which can originate from blood monocytes and resident yolk sac-derived macrophages, perform functions far beyond the classical tasks of phagocytosis and antigen presentation. As a major source of cytokines, chemokines, and enzymes, TAMs modulate the tumor microenvironment, supporting processes such as angiogenesis, invasion, metastasis, and EMT. Particularly, TAMs exhibiting the M2 phenotype, which dominate in the advanced stages of ovarian cancer, are key promoters of EMT and thus play an important role in the loss of epithelial features and acquisition of mesenchymal features by tumor cells. M2 macrophages support EMT by secreting cytokines and chemokines, including TGF-β, IL-10, and CCL18. TGF-β activates the Smad2/3 pathway, which initiates changes in tumor cell gene expression, leading to the loss of E-cadherin, increased vimentin expression, and increased cell invasiveness. CCL18 induces the transcription factor ZEB1, which regulates EMT-related genes and simultaneously promotes M-CSF secretion, which enhances the recruitment and polarization of TAMs toward the M2 phenotype. IL-10 further suppresses the immune response of T lymphocytes in the tumor microenvironment while promoting EMT by reducing the activity of inhibitory factors such as miR-200 [85,86]. In hypoxic conditions, common in ovarian cancer, M2 TAMs produce IL-1β, which activates HIF-1α in tumor cells, creating a feedback loop that enhances EMT. HIF-1α increases the expression of genes such as *SNAIL*, *SLUG*, and *TWIST*, which are crucial for mesenchymal transition, and promotes angiogenesis and tumor adaptation to hypoxia (Table 2) [87,88,89,90].

An additional mechanism supporting EMT by TAMs is activating the Wnt/β-catenin pathway through the secretion of the ligand Wnt5a. Wnt5a, which is also produced by mesothelial cells in neoplastic ascites, promotes EMT by activating the TGF-β1/Smad2/3 and Hippo-YAP1/TAZ-TEAD pathways. *ZEB1*, a key regulator of EMT, is activated in cancer cells by CCL18 secreted by TAMs, leading to the increased expression of genes involved in extracellular matrix degradation, such as MMP9, and promotes the recruitment of additional TAMs by inducing CCL2 [91,92,93].

M2 macrophages can also promote EMT via the EGF signaling pathway. EGF production by TAMs activates the EGFR receptor in tumor cells, promoting their migration and invasiveness. Blockade of EGFR with inhibitors such as *AG1478* reversed EMT in experimental studies, confirming the importance of this pathway in tumor progression. Furthermore, the long non-coding RNA LIMT expression in tumor cells showed the ability to inhibit TAM-induced EMT, reducing tumor size and ascites in animal models, indicating the potential use of lncRNAs in ovarian cancer therapy [87].

To conclude, the clinical significance of TAM-induced EMT is well documented, and growing evidence points to possible diagnostic and therapeutic consequences of this phenomenon. A higher ratio of M1 to M2 macrophages correlates with a better prognosis, whereas the dominance of M2 macrophages, especially those expressing CD163, is associated with poorer overall and progression-free survival. Selective depletion of CD163+ Tim4+ macrophages in animal models led to the inhibition of EMT, which underlines their role in ovarian cancer progression. TAMs have also been shown to be involved in omental metastasis, which supports their role in tumor cell dissemination. In summary, TAMs, especially those of the M2 phenotype, are a key element of the tumor microenvironment, promoting EMT and supporting tumor adaptation to changing conditions. These mechanisms open new therapeutic possibilities, such as the repolarization of TAMs to the M1 phenotype, blockade of TAM-secreted factors, or the use of lncRNAs, which may significantly improve the prognosis of ovarian cancer patients [94,95,96,97].

#### 3.3.2. TILs in Ovarian Cancer

Tumor-infiltrating lymphocytes (TILs) play an essential role in the TME of ovarian cancer, influencing tumor progression, immune response, and the process of EMT. TILs are defined as CD3+ T lymphocytes that infiltrate tumor margins, and their presence is usually associated with a favorable prognosis. However, their role in EMT is more complex and may differ depending on the subpopulations, which can be both pro- and antitumor [98,99].

TILs can influence EMT by modulating the tumor microenvironment via cytokines, chemokines, and interactions with other TME cells. For example, CD8+ lymphocytes, crucial effectors of antitumor cytotoxicity, are associated with EMT inhibition. Their presence in the tumor correlates with less invasiveness of tumor cells and better prognosis, as confirmed by a study by Hwang et al. [100]. CD8+ TILs, through secretion of IFN-γ, can inhibit EMT-related pathways, such as TGF-β/Smad, and regulate the expression of genes crucial for maintaining the epithelial phenotype, such as E-cadherin. Additionally, the presence of CD8+ TILs is associated with significant infiltration of M1-polarized macrophages, which also inhibit EMT and thus promote better control of tumor progression [101,102,103].

In contrast, CD4+ lymphocytes, depending on their phenotype, may have a more diverse effect on EMT. CD4+ Treg lymphocyte subsets, often present in the tumor, promote EMT by secreting IL-10 and TGF-β, which activate signaling pathways supporting the loss of epithelial features and acquisition of a mesenchymal phenotype by tumor cells. Tregs can also suppress the antitumor activity of other lymphocytes, including CD8+, which supports the EMT process and promotes tumor aggressiveness [104,105,106].

Characterization of TILs in ovarian cancer may also be relevant in the context of tumor immunological classification. Immunologically “cold” tumors, with low lymphocyte infiltration, are often associated with advanced EMT changes and more excellent resistance to immunotherapy [107,108]. As shown in the literature, “cold” tumors can be divided into two patterns: quantitatively “cold”, where dysfunctional T lymphocytes are present, and qualitatively “cold”, dominated by tumor-nonspecific lymphocytes. Primary lesions usually exhibit quantitatively “cold” patterns, whereas a qualitatively “cold” phenotype more often characterizes omental metastases. In both cases, the presence of EMT is intensified by the lack of an effective lymphocytic response (Table 2) [109].

In addition, there is evidence for the involvement of specific TIL subpopulations in regulating EMT. For example, the expression of the CD39 molecule on lymphocytes is associated with T cell exhaustion and poorer prognosis in HGSC. Exhausted CD39+ T cells are characterized by the loss of effector functions, which promotes EMT progression and further tumor aggressiveness. Interestingly, subsets of these cells express markers typical of tissue-resident memory T cells, which generally play a key role in the antitumor immune response. Their exhaustion and loss of function in the context of ovarian cancer emphasize the importance of TILs in the dynamic regulation of EMT [110,111,112].

#### 3.3.3. Th1, Th2, Th17, Th9, and Th22 Balance in Ovarian Cancer

The balance among Th1, Th2, Th17, Th9, and Th22 lymphocyte subpopulations plays a key role in regulating EMT in ovarian cancer, influencing the dynamics of the TME and tumor progression. The cytokines that are secreted by different lymphocyte subpopulations modulate EMT, leading to increased invasiveness, migration, and metastatic capacity of tumor cells. An imbalance towards the dominance of EMT-supporting subpopulations, such as Th2, Th17, and Th22, while weakening the function of Th1 and Th9, promotes ovarian cancer progression and correlates with a poorer prognosis (Table 2) [113,114].

Th1 lymphocytes play a key antitumor role by secreting pro-inflammatory cytokines, such as interferon gamma (IFN-γ) and interleukin 2 (IL-2), which enhance the cytotoxic response of CD8+ lymphocytes and the activity of M1 macrophages. IFN-γ inhibits EMT by regulating the TGF-β/Smad and Wnt/β-catenin pathways, limiting the loss of E-cadherin and the acquisition of mesenchymal features by tumor cells. Th1 cytokines also support the preservation of epithelial markers, such as cytokeratin, which reduces tumor cells’ invasiveness and metastatic capacity. Th1 deficiency in the tumor microenvironment is commonly observed in advanced stages of ovarian cancer, which correlates with the severity of EMT and poorer prognosis (Table 2) [115,116]

In contrast to Th1, Th2 lymphocytes support EMT by secreting anti-inflammatory cytokines such as interleukin 4 (IL-4), interleukin 5 (IL-5), and interleukin 13 (IL-13). IL-4 activates the STAT6 pathway, which regulates EMT-related genes, increasing the expression of mesenchymal markers such as vimentin and N-cadherin and supporting tumor cell migration. Th2 cytokines also promote macrophage polarization to the M2 phenotype, which enhances the secretion of TGF-β and IL-10, key regulators of EMT. Th2 dominance in the tumor microenvironment is often associated with advanced EMT progression, increased tumor invasiveness, and treatment resistance (Table 2) [117,118].

**Figure 5:**
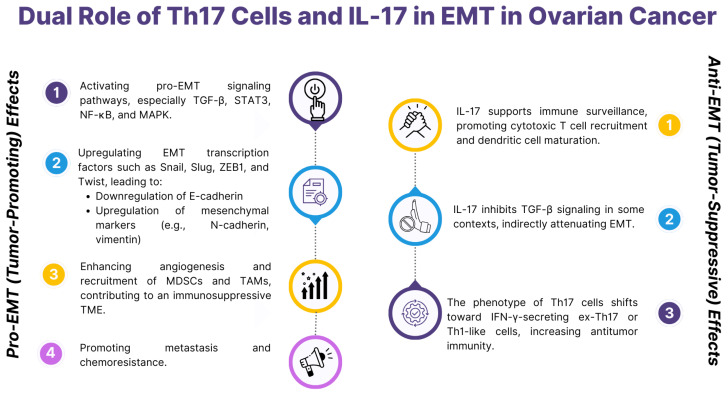
Dual role of Th17 and IL-17 in EMT in ovarian cancers, based on [119,120,121,122].

Th17 lymphocytes, secreting interleukin 17 (IL-17), play a dual role in ovarian cancer EMT, which depends on the tumor microenvironment and interactions with other cytokines. IL-17 activates signaling pathways such as NF-κB, PI3K/AKT, and STAT3, which are associated with EMT. Their activation leads to increased mesenchymal markers such as vimentin and N-cadherin and decreased expression of epithelial markers such as E-cadherin. As a result of these changes, tumor cells can migrate and invade, which supports tumor progression and its ability to colonize distant tissues. Additionally, IL-17 can regulate the expression of EMT transcription factors such as *SNAIL*, *SLUG*, and *TWIST*, enhancing the transition to a mesenchymal phenotype. The role of Th17 cells in EMT is ambivalent and may vary depending on the tumor microenvironment. In some contexts, IL-17 acts protumorigenically by promoting angiogenesis and the expression of immunosuppressive cytokines such as IL-10 and TGF-β, which support EMT and suppress the immune response. On the other hand, IL-17 can enhance the antitumor response by recruiting neutrophils, macrophages, and other immune effector cells that support tumor cell elimination. In addition, IL-17 induces the production of pro-inflammatory cytokines such as IL-6, TNF-α, and GM-CSF, which may modulate the TME in favor of an antitumor response. Depending on the context, Th17 interacts with other cells in the TME to enhance or suppress EMT. For example, IL-17 can stimulate CAFs to secrete proangiogenic factors such as VEGF and extracellular matrix supporting EMT. On the other hand, IL-17 can support the recruitment and activation of Th1 and CD8+ lymphocytes, which are crucial for antitumor immune responses and can inhibit EMT by inhibiting signaling pathways supporting mesenchymal transition [119,120,121,122] (Table 2 and Figure 5).

Th9 lymphocytes, which secrete interleukin 9 (IL-9), potentially benefit from counteracting EMT in ovarian cancer. IL-9 enhances the activity of CD8+ lymphocytes and M1 macrophages, which inhibit EMT and support the maintenance of the epithelial phenotype of cancer cells. In addition, IL-9 can inhibit the TGF-β/Smad pathway, which limits the loss of E-cadherin and reduces cancer cell migration. Although the role of Th9 in EMT in ovarian cancer is relatively poorly understood, the presence of these cells in the TME is associated with better prognosis and lower tumor aggressiveness. On the other hand, Th9 accumulation in tumor tissue might induce EMT and metastasis through its main central effector cytokine IL-9, as was presented in the case of lung cancer (Table 2) [123,124,125,126].

Th22 cells, which secrete interleukin 22 (IL-22), have a variable role in regulating EMT depending on the tumor microenvironment. IL-22 promotes EMT by activating the JAK/STAT3 pathway, which regulates the expression of genes associated with the mesenchymal phenotype, such as *SNAIL* and *TWIST*. At the same time, IL-22 can promote angiogenesis, which supports the migration and metastatic capacity of tumor cells. However, in specific contexts, IL-22 can act protectively, supporting tissue regeneration and limiting tumor progression. The role of Th22 in EMT requires further study. Still, there is evidence to suggest that its effect is strongly dependent on the presence of other cytokines and interactions with other lymphocyte subpopulations (Table 2) [127,128].

**Table 2:** Comparison of the functions of selected immune cells in EMT in ovarian cancer.

Cell Type	Subtype	Role in EMT	Key Cytokines/Factors	Mechanisms of Action	Prognostic Implication	References
Macrophages	M1	Inhibit EMT	IL-12, TNF-α	Promote inflammation and cytotoxicity; oppose immunosuppressive signals; inhibit TGF-β signaling; enhance antigen presentation.	Higher M1/M2 ratio is linked to better clinical outcomes.	[85,86,87,88,89,90,91,92,93,94,95,96,97]
	M2 (TAMs)	Promote EMT	TGF-β, IL-10, CCL18, IL-1β,	Activate Smad2/3, Wnt/β-catenin, EGFR, Hippo-YAP1/TAZ, and PI3K/AKT pathways; induce ZEB1, TWIST, and SLUG; increase MMP9 and CCL2; support angiogenesis and hypoxia.	Correlated with poor survival; CD163^+^Tim4^+^ TAMs drive EMT and metastasis.	
T cells	CD8+ cytotoxic T cells	Inhibit EMT	IFN-γ	Suppress TGF-β/Smad pathway; maintain E-cadherin and cytokeratin expression; indirectly enhance M1 macrophage recruitment.	Associated with less invasive tumors and better survival.	[100,101,102,103]
T cells	Tregs (CD4^+^)	Promote EMT	IL-10, TGF-β	Suppress CD8^+^ T cell activity; induce EMT-supportive signaling; promote immunosuppression and tumor progression.	Linked to poor prognosis and tumor immune evasion.	[104,105,106]
	CD39^+^ exhausted T cells	Promote EMT	-	Exhibit loss of effector function; express markers of tissue-resident memory T cells; fail to respond to tumor antigens, allowing EMT progression.	Indicate poor immunosurveillance and are associated with worse outcomes.	[110,111,112]
Th cells	Th1	Inhibit EMT	IFN-γ, IL-2	Inhibit TGF-β/Smad and Wnt/β-catenin pathways; preserve epithelial markers (E-cadherin, cytokeratin); stimulate M1 and CD8^+^ activity.	Protective; Th1 deficiency correlates with advanced EMT and worse prognosis.	[115,116,117,118]
	Th2	Promote EMT	IL-4, IL-5, IL-13	Activate the STAT6 pathway; upregulate vimentin and N-cadherin; promote M2 polarization; reinforce EMT signals.	Linked to aggressive tumors and therapy resistance.	
	Th17	Dual (context-dependent)	IL-17	Activate NF-κB, PI3K/AKT, and STAT3; increase mesenchymal markers (vimentin, N-cadherin); modulate angiogenesis and immunosuppression or enhance immune effectors.	Role varies with the TME context; it may promote or inhibit EMT depending on the cytokine milieu.	[119,120,121,122]
	Th9	Inhibit EMT (potentially)	IL-9	Stimulate CD8^+^ and M1 cells; suppress TGF-β/Smad signaling; may reduce EMT and tumor aggressiveness, though conflicting roles have been observed in other cancers.	Presence suggests favorable prognosis; therapeutic potential under investigation.	[123,124,125,126]
	Th22	Promote EMT	IL-22	Activate JAK/STAT3; induce EMT-related transcription factors (SNAIL, TWIST); support angiogenesis and cell migration; occasionally support tissue repair.	Strongly dependent on tumor microenvironment; mostly pro-EMT in ovarian cancer.	[127,128]

### 3.4. Recent Evidence on the Role of EMT in the Tumor Biology of Ovarian Cancer

EMT is a key biological process that plays a significant role not only in the progression and metastasis of ovarian cancer but also in the adaptation of neoplastic cells to the dynamic tumor microenvironment. Ongoing research binds various stages of ovarian tumorigenesis and the concept of cancer stem cell-like cells (CSCs) to EMT [129].

There is convincing evidence that EMT is active in the early stages of neoplasia, suggesting that this process plays a role in initiating tumorigenesis. EMT markers are regularly detected in premalignant noninvasive lesions, such as fallopian tube intraepithelial lesions. Of particular note is the marker CD44, which is also considered a potential marker of CSC. A study by Takahashi et al. [130], showed that CD44 can activate EMT by interacting with the CD44–moesin–actin complex. A subsequent study by Alwosaibai et al. [131], revealed an inverse relationship between CD44 expression and PAX2 protein levels in ovarian cancer cells. PAX2, a protein that regulates epithelial cell differentiation during embryogenesis and postovulatory repair, is frequently mutated in secretory outgrowths (SCOUT) and serous fallopian tube intraepithelial carcinoma (STIC). The presence of CD44+ in the distal fallopian tube epithelium strengthens the hypothesis of a fallopian tube origin of HGSC. Additionally, targeting CD44 with miR-199a has effectively reduced tumor growth in in vitro and in vivo studies, making CD44 an attractive therapeutic target [130,131,132,133,134,135].

Recent studies have highlighted the important role of EMT-related lncRNAs in regulating these oncogenic processes in ovarian cancer. Long non-coding RNAs (lncRNAs) are RNA molecules exceeding 200 nucleotides in length that do not encode proteins but perform key regulatory functions in various biological processes, including EMT. EMT-related lncRNAs can act as oncogenes or tumor suppressors by influencing various molecular pathways. For example, lncRNA *HOTAIR* promotes EMT in ovarian cancer by modulating EMT markers’ expression, which increases cancer cells’ ability to invade and metastasize. Similarly, lncRNA *MALAT1* is associated with increased EMT and poor prognosis in ovarian cancer patients. On the other hand, some lncRNAs, such as *MEG3*, act as tumor suppressors by inhibiting EMT and limiting tumor progression. The regulatory mechanisms of EMT-related lncRNAs in ovarian cancer are diverse. LncRNAs can act as competitive endogenous RNAs (ceRNAs), capturing microRNAs (miRNAs) and regulating the expression of EMT-related genes. Additionally, lncRNAs can interact with chromatin-modifying complexes to influence gene expression at the epigenetic level. For example, *HOTAIR* can recruit the *PRC2* complex to specific gene promoters, leading to histone modifications that promote EMT. Understanding the role of EMT-related lncRNAs in ovarian cancer is of significant clinical importance. LncRNAs show potential as diagnostic and prognostic biomarkers due to their specific tissue expression and stability in body fluids. Furthermore, targeting EMT-related lncRNAs may provide novel therapeutic strategies to inhibit EMT, limit metastasis, and overcome drug resistance in ovarian cancer [136].

Although not always highlighted, EMT should not be conceptualized as a linear process; intermediate “EMT hybrid” (E/M) cells may play an even more critical role in tumor progression than purely epithelial or mesenchymal cells. A study by Strauss et al. has demonstrated the presence of transitional E/M hybrid cells in situ in ovarian cancer, underlining their importance in tumor development. They found that all CD133+ cells, which are considered a marker of CSCs, had features of the E/M phenotype, underlining the crucial link between the EMT transition and cancer stem cells. E/M hybrid cells are characterized by high phenotypic plasticity and the ability to adapt to the variable tumor microenvironment, making them particularly difficult to target in therapy [137].

In addition, EMT studies suggest a link between intermediate steps of EMT and the formation of premetastatic niches that support tumor cell colonization and metastasis. This process highlights the importance of dynamic interactions between the tumor microenvironment and tumor cells. The importance of EMT in ovarian cancer goes beyond its role in migration and invasion; it also plays a central role in resistance to therapies and regulation of the immune response, making it a priority target for new therapeutic strategies. Understanding the detailed mechanisms of EMT, including the role of E/M hybrid cells, is a significant challenge in oncology. Still, at the same time, it offers hope for more precise and effective methods of treating ovarian cancer [138,139,140,141].

### 3.5. The Importance of EMT in Ovarian Cancer Chemotherapy

**Figure 6:**
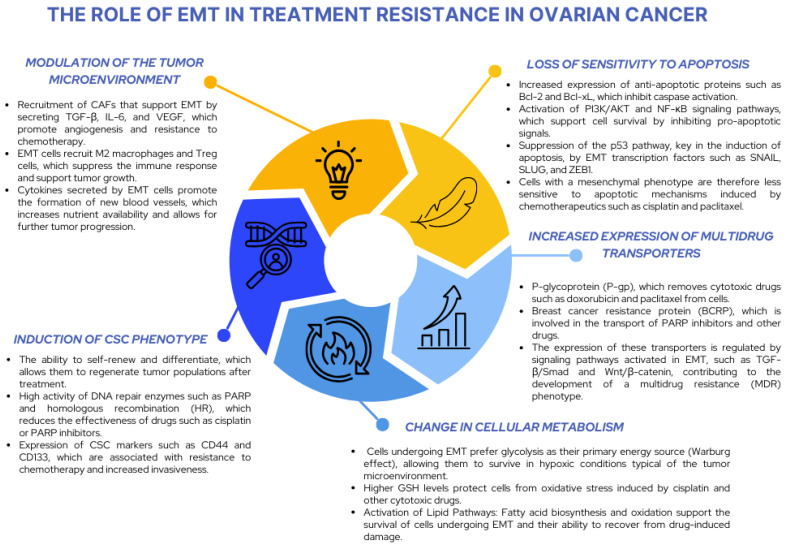
The role of EMT in treatment resistance in ovarian cancer [142,143,144,145,146,147,148,149].

EMT plays a key role in chemotherapy resistance in ovarian cancer by influencing the ability of cancer cells to evade apoptosis, adapt to the TME, and develop a multidrug resistance (MDR) phenotype. By reprogramming the phenotype of cancer cells, EMT allows them to acquire mesenchymal characteristics, such as motility, invasiveness, resistance to oxidative stress, and altered metabolic pathways, which are key to evading the effects of chemotherapeutic agents (Figure 6).

The EMT process leads to molecular reprogramming that reduces the sensitivity of cells to apoptosis while increasing the expression of antiapoptotic proteins such as Bcl-2 and Bcl-xL. The activation of PI3K/AKT signaling pathways and suppression of the p53 pathway by EMT transcription factors such as *SNAIL* and *ZEB1* further enhance the ability of cancer cells to evade chemotherapy-induced apoptotic mechanisms. Furthermore, EMT promotes the development of the MDR phenotype by regulating ABC transporters such as P-gp and BCRP, which actively remove chemotherapeutic agents from cells. This mechanism is supported by the activation of TGF-β/Smad pathways and reorganization of the plasma membrane, which limit drug penetration. EMT cells also undergo metabolic reprogramming that supports their survival under chemotherapy-induced stress. They change their energy sources, switching to aerobic glycolysis (Warburg effect), which allows for better adaptation to hypoxic conditions in the tumor microenvironment. Additionally, they increase the production of glutathione (GSH), which protects them from oxidative stress, and change lipid pathways, supporting the biosynthesis and oxidation of fatty acids, increasing their resistance to chemotherapy [142,143,144].

EMT in ovarian cancer is closely associated with interactions with the tumor microenvironment, including CAFs, M2 macrophages, and Treg cells. Cells undergoing EMT secrete cytokines and growth factors, such as TGF-β, IL-6, and VEGF, which support angiogenesis, immunosuppression, and the formation of protective niches within the tumor. CAFs support EMT by providing extracellular matrix components and modulating signaling pathways that support mesenchymal transition. Furthermore, M2 macrophages and Treg cells in the tumor microenvironment promote EMT and related processes, such as invasiveness and metastasis [145,146,147].

As was previously stated, EMT is also associated with the acquisition of a CSC phenotype that is particularly resistant to chemotherapy. These cells are characterized by the ability to self-renew, the high activity of DNA repair enzymes, and the expression of markers such as CD44 and CD133, which support their survival and ability to adapt to a changing environment. The presence of these cells in the tumor is associated with an aggressive course of the disease and resistance to many therapies [147,148].

The influence of EMT on the efficacy of specific chemotherapeutic therapies is particularly evident in the case of drugs such as cisplatin, paclitaxel, and PARP inhibitors. Cells subjected to EMT are more resistant to cisplatin due to increased activity of DNA repair systems such as homologous recombination (HR). In the case of paclitaxel, changes in the cytoskeleton of mesenchymal cells limit the drug’s efficacy, which stabilizes microtubules. EMT cells are also less sensitive to PARP inhibitors, which results from activating alternative DNA repair mechanisms [149].

One of the key mechanisms linking EMT to tumor resistance to immunotherapy is the increased expression of immune checkpoint molecules such as PD-L1. EMT has been shown to promote PD-L1 expression on the surface of tumor cells, inhibiting cytotoxic T cell activity and facilitating tumor progression [150]. The overexpression of PD-L1 plays a crucial role in the mechanisms of immune evasion by CD8^+^ lymphocytes, which is confirmed by studies showing that tumors subjected to EMT are characterized by significantly higher PD-L1 expression [151].

In the context of ovarian cancer, the relationship between EMT status and immunotherapy efficacy is becoming increasingly well documented. According to the results of Li et al., patients with a higher EMT index show reduced efficacy of checkpoint inhibitors and limited tumor infiltration by T lymphocytes [115]. EMT contributes to the formation of the TME, including through the recruitment of Tregs and MDSCs, inhibiting the antitumor response [152].

Of particular importance in this context is the TGF-β pathway, considered one of the key inducers of EMT. The activation of this pathway triggers several mechanisms leading to the development of immunosuppression in ovarian cancer [153], including the recruitment of TAMs, which enhance the tumor’s ability to evade immune surveillance [115].

These changes have important clinical implications for the efficacy of immunotherapy. Although checkpoint inhibitors such as anti-PD-1 and anti-PD-L1 antibodies demonstrate high efficacy in many cancers, their efficacy in ovarian cancer remains limited. According to data published by Su et al., response rates to these therapies in this group of patients reach only 11.5–15%, which is lower compared to other types of cancer [154].

Similar limitations also apply to vaccine strategies. Reactive changes occurring in the TME, including the accumulation of immunosuppressive factors, effectively limit the recruitment and activation of T lymphocytes specific for tumor antigens [155]. For example, the presence of IL-10 and TGF-β in the ascites of ovarian cancer patients is a clear example of environmental inhibition of post-vaccination response [156].

Current clinical trials focus on combination strategies, in which ICIs are used together with chemotherapy or other standard drugs to overcome EMT-related resistance. Hartl et al. showed that combining chemotherapy with T cell-enhancing immunotherapy can significantly improve survival in preclinical models of ovarian cancer [157]. These results are consistent with reports suggesting that combination therapies can more effectively reverse the immunosuppressive properties of the TME [158]. Promising effects have also been observed in studies using niraparib (PARP inhibitor) in combination with pembrolizumab (anti-PD-1 antibody), which showed higher efficacy than standard therapies [159]. Similar results have been obtained in studies combining bevacizumab with checkpoint inhibitors, indicating the potential of these strategies in the treatment of recurrent and metastatic ovarian cancer [160]. Growing evidence suggests that exosomes are essential in promoting EMT and immune evasion in ovarian cancer. Studies by Cai et al. have shown that exosomes secreted by TGF-β1-treated fibroblasts can carry specific microRNAs that induce the expression of EMT-related genes in cancer cells [161]. This indicates the necessity of considering exosomal communication in the TME as a potential therapeutic target, especially in improving the response to immunotherapy. Despite the promising results of combination therapies, effective targeting of EMT and its associated immune evasion mechanisms remains challenging. Liu et al. emphasize that moderate response rates to ICIs require developing new strategies that simultaneously enhance the T cell response and break EMT-driven immunosuppression [162]. It is also crucial to better understand the role of tumor stroma components—such as tumor-associated macrophages and fibroblasts—in modulating the immune response and regulating EMT [163]. An additional challenge is the concern about the occurrence of so-called hyperprogressive disease in response to immunotherapy, which may be of particular importance in patients with advanced ovarian cancer exhibiting features of EMT [164]. This phenomenon underscores the need to develop comprehensive biomarkers that will allow for the precise tailoring of treatment to the individual characteristics of a given patient’s TME and EMT status.

Therefore, therapeutic strategies aimed at inhibiting EMT may be a promising way of improving the efficacy of immunotherapy in ovarian cancer. The reduction in EMT activity may lead to the transformation of the TME into a more immunogenic and protumor response. For this purpose, TGF-β pathway inhibitors and transcription factors regulating EMT are considered. Moreover, combining immunotherapy with targeted therapies in EMT may demonstrate a synergistic effect, improving the treatment’s efficacy and limiting the tumor phenotype’s aggressiveness [165].

## 4. Further Research on EMT in the Development, Progression, and Treatment of Ovarian Cancer

### 4.1. EMT Transcription Factors and Apoptosis Resistance and Multidrug Resistance

Understanding the mechanisms of EMT opens new research directions that can contribute to more effective diagnostic, prognostic, and therapeutic strategies. One of the priority areas of research is the identification of EMT signaling pathways, such as TGF-β/Smad, Wnt/β-catenin, and Hip-po/YAP/TAZ. These pathways regulate the plasticity of cancer cells and affect their ability to metastasize and adapt in the tumor microenvironment. Interactions between EMT and inflammatory processes in the tumor, including the role of inflammasomes, also require further investigation. It is also essential to deepen the knowledge of transcription factors, such as *SNAIL*, *SLUG*, *ZEB1*, *ZEB2*, and *TWIST*, which modulate EMT, influencing the ability of cancer cells to avoid apoptosis and the development of the MDR phenotype [166,167,168].

The TME plays a key role in the regulation of EMT. Studies should focus on interactions between cancer cells, CAFs, M2 macrophages, and Treg lymphocytes. These cells secrete cytokines such as TGF-β, IL-6, and VEGF, which support EMT and promote ovarian cancer progression. As one of the main factors of the TME, hypoxia supports EMT by activating HIF-1α, which promotes the formation of premetastatic niches. Equally important are immunological interactions, including the influence of immune cells such as Th17 lymphocytes, which can both support and inhibit EMT depending on the local microenvironmental conditions. Due to the complex effect of Th17 on EMT, the modulation of this lymphocyte subpopulation is a potential therapeutic target in ovarian cancer. Studies indicate that dendritic vaccines inducing a Th17 response can effectively stimulate an antitumor response in preclinical models of ovarian cancer. Moreover, IL-17 is important in improving the efficacy of combination therapies, such as immune checkpoint inhibitors, even in cases of primary or acquired resistance to these therapies. On the other hand, IL-17 blockade in selected cases could limit EMT and tumor aggressiveness, suggesting a bi-pronged therapeutic approach depending on the characteristics of the TME. Likewise, Th9 cells’ effect on the induction of EMT might be countered by the anti-neoplastic effects of their cytokines, which might be used in selective targeting of specific signaling pathways [115,116,117,118,119,120,121,122,123,124,125,126,127,128].

### 4.2. The Relationship Between EMT and the Cancer Stem Cell (CSC) Phenotype and Resistance Mechanisms

There is a close relationship between EMT and the acquisition of cancer stem cell (CSC) characteristics. During EMT, epithelial cells undergo reprogramming, which often coincides with the stem cell phenotype; they demonstrate the ability to self-renew, slowly divide, and resist many therapies. In ovarian cancer, several markers characteristic of cells with stem cell characteristics have been identified: these include, among others, the surface proteins CD44 and CD133 (Prominin-1) and high activity of the enzyme ALDH1 (aldehyde dehydrogenase). CD133^+^/ALDH^+^ cells isolated from ovarian tumors showed the ability to initiate new tumors (high oncogenic potential) and at the same time were characterized by the EMT transcriptional profile; they had increased invasiveness and expression of vimentin and N-cadherin. This suggests that EMT promotes the emergence of a CSC-like population. Conversely, many CSCs can spontaneously adopt mesenchymal features. For example, ALDH1-bright ovarian cancer cells show increased migration, invasiveness, and resistance to apoptosis—typical attributes of EMT cells. Such phenotypic plasticity is a therapeutic challenge: even if most tumor cells are destroyed, the remaining EMT/CSC cells can give rise to disease relapse. One of the resistance mechanisms associated with this phenotype is the overexpression of ABC transporters (ATP-binding cassette). Cells after EMT often show an increased expression of P-glycoprotein (ABCB1, P-gp) or MRP1-5 (ABCC) or BCRP (ABCG2) proteins, which actively remove drugs from cells, causing multidrug resistance (MDR). It has been shown that the expression of ABC genes changes dynamically during EMT; as epithelial features are lost, there is a gradual increase in the level of efflux pumps protecting the cell from cytostatics. Additionally, EMT/CSC cells can use DNA repair mechanisms to survive chemotherapy-induced damage (e.g., the aforementioned regulation by ZEB1) and switch metabolism to a more favorable one under stress conditions (e.g., aerobic glycolysis or use of alternative energy sources). In clinical practice, it has been found that recurrent ovarian tumors often show increased levels of CSC and EMT markers compared to primary tumors, which is associated with resistance to standard platinum drugs and taxanes. Therefore, treatment strategies increasingly target these populations; studies are underway on inhibitors of pathways maintaining CSC self-renewal (e.g., Notch, Hedgehog, Wnt), immunotherapy directed against CSC markers (e.g., anti-CD133), and desensitization of ABC pumps (P-glycoprotein inhibitors). Significantly, eliminating the EMT/CSC phenotype or inducing differentiation of these cells to a more epithelial state could significantly improve the efficacy of treatment and reduce the risk of relapse [168,169,170,171].

### 4.3. EMT–MET Plasticity in Metastasis: Significance and Therapeutic Implications

EMT is a reversible process; cells that have undergone a mesenchymal transition retain the ability to reacquire epithelial features through MET (mesenchymal–epithelial transition). Such EMT–MET plasticity is crucial for the complete success of the metastasis process. In the metastasis model proposed by Jean-Paul Thiery as early as 2002, cancer cells must undergo EMT to leave the primary tumor and migrate to distant tissues but then undergo MET in a new habitat to organize the growth of a secondary tumor [172]. In the case of ovarian cancer, whose metastases often develop in the peritoneum and omentum, it is observed that cells present in the ascitic fluid (circulating in the abdominal cavity) show the EMT marker phenotype. In contrast, cells in secondary foci can partially regain epithelial markers. Intermediate states (partial EMT) further facilitate this adaptation; E/M hybrid cells can both circulate and efficiently colonize a new site, depending on environmental signals. However, this plasticity is a double-edged sword from a therapeutic perspective: blocking EMT at the primary tumor stage may paradoxically retain cells in an epithelial state, which form large solid tumors but are more sensitive to drugs; on the other hand, allowing EMT allows cells to disperse but may later enable MET and metastatic growth. Therefore, different therapeutic approaches targeting plasticity are being discussed: inhibiting both EMT and MET (so that circulating cells remain quiescent and do not establish colonies) or, on the contrary, pharmacologically enforcing MET before cells reach the metastatic niche, making them more sensitive to anoikis (death after loss of connections) and immune attack. For now, such interventions are at the preclinical stage. One clue is that the partial EMT state seems to be the most oncogenic; searching for molecules that would “lock” the cell in one state is ongoing. For example, activating certain epigenetic factors can block the ability to undergo MET, which would lock circulating cells in the mesenchymal state, depriving them of the ability to metastasize (but such cells can remain latent for a long time). Alternatively, anti-adhesion therapies in target niches are being considered—since colonization requires the re-formation of epithelial connections, integrin or cadhedrin inhibitors at the site of potential metastasis could impede the settlement of incoming cells. From a clinical point of view, single-cell examination of circulating tumor cells (CTCs) and cells in ascitic fluid allows for assessment of these cells’ EMT/MET stage and prediction of the risk of metastasis. EMT/MET plasticity is therefore a central point of metastasis and a target for new anti-metastatic strategies: an ideal therapy should either prevent both processes (EMT and MET) or manipulate them so that cancer cells are “trapped” in non-survivable states [17,138,173].

The role of EMT in ovarian cancer metastasis also requires further investigation. The mechanisms by which EMT promotes metastatic colonization, including E/M cell plasticity and EMT-to-MET (epithelial transition) reversal, may provide valuable information on the ability of cells to adapt in distant tissues. Understanding these processes is crucial for developing targeted therapies limiting tumor dissemination. In the therapeutic area, EMT inhibitors, such as anti-TGF-β antibodies, Hippo/YAP/TAZ pathway modulators, or Wnt/β-catenin inhibitors, are particularly promising. Combining such therapies with immune checkpoint inhibitors (PD-1/PD-L1) or chemotherapy may significantly improve treatment efficacy. The use of new technologies, such as single-target RNA sequencing (scRNA-seq), may provide more detailed information on the heterogeneity of cells subjected to EMT and their interactions with the TME. Three-dimensional cell culture models and ovarian cancer organoids that more faithfully represent in vivo conditions may be essential tools for studying EMT and testing new therapies.

## Figures and Tables

**Table 1:** Comparative summary of EMT types 1, 2, and 3 [64].

Feature	EMT Type 1	EMT Type 2	EMT Type 3
Primary Context	Embryogenesis and organ development	Tissue regeneration and wound healing	Tumor progression and metastasis
Physiological or Pathological Role	Physiological; tightly regulated	Both physiological and pathological, depending on context	Pathological; associated with malignancy
Triggering Factors	Developmental signals (e.g., Wnt, FGF, Notch)	Inflammatory cytokines, growth factors (e.g., TGF-β, IL-6), tissue injury	Oncogenic signals, hypoxia, TGF-β, tumor microenvironment components
Cellular Outcome	Formation of mesenchymal progenitor cells from epithelial precursors; essential for neural crest formation, heart development, etc.	Transient mesenchymal conversion to promote fibrosis resolution and tissue remodeling	Acquisition of mesenchymal traits by epithelial tumor cells, leading to increased motility, invasiveness, and therapy resistance
Molecular Features	Downregulation of E-cadherin, upregulation of N-cadherin, *SNAI1*, *SNAI2* (developmentally regulated)	Similar molecular profile as type 3 but context-dependent and reversible under normal conditions	Persistent activation of EMT-TFs (*SNAI1*, *SNAI2*, *ZEB1*, *ZEB2*, *TWIST1*), stable repression of epithelial genes, activation of invasion/metastasis programs
Outcome in Cancer	Not associated with neoplastic transformation	May indirectly promote tumorigenesis in chronic inflammation via fibrosis and immunosuppression	Directly contributes to carcinogenesis, epithelial plasticity, intravasation, metastasis, and chemoresistance
Relevance to Ovarian Cancer	Not applicable	Chronic peritoneal inflammation may contribute to EMT induction and tumor-supportive stroma	Crucial in promoting dissemination of ovarian cancer cells within the peritoneal cavity, enhancing metastatic potential and resistance to platinum-based chemotherapy

## Data Availability

All data supporting the findings of this study are available from the first author of the manuscript upon reasonable request.
